# Crystal structure, Hirshfeld surface analysis, inter­molecular inter­action energies, energy frameworks and DFT calculations of 4-amino-1-(prop-2-yn-1-yl)pyrimidin-2(1*H*)-one

**DOI:** 10.1107/S2056989023009933

**Published:** 2023-11-21

**Authors:** Mouad Lahyaoui, Amal Haoudi, Badr Eddine Kartah, Ahmed Mazzah, Tuncer Hökelek, Joel T. Mague, Youssef Kandri Rodi, Nada Kheira Sebbar

**Affiliations:** a Laboratoire de Chimie Organique Appliquée, Université Sidi Mohamed Ben Abdallah, Faculté des Sciences et Techniques, Route d’Immouzzer, BP 2202 Fez, Morocco; b Laboratory Of Applied Organic Chemistry, Sidi Mohamed Ben Abdellah University, Faculty Of Science And Technology, Road Immouzer, BP 2202 Fez, Morocco; c Laboratory of Plant Chemistry, Organic and Bioorganic Synthesis, Faculty of Sciences, Mohammed V University in Rabat, 4 Avenue Ibn Battouta, BP 1014 RP, Morocco; d Science and Technology of Lille USR 3290, Villeneuve d’ascq cedex, France; e Department of Physics, Hacettepe University, 06800 Beytepe, Ankara, Türkiye; f Department of Chemistry, Tulane University, New Orleans, LA 70118, USA; g Laboratoire de Chimie Organique Appliquée, Université Sidi Mohamed Ben Abdallah, Faculté des Sciences et Techniques, Route dImmouzzer, BP 2202 Fez, Morocco; h Laboratory of Organic and Physical Chemistry, Applied Bioorganic Chemistry Team, Faculty of Sciences, Ibn Zohr University, Agadir, Morocco; Vienna University of Technology, Austria

**Keywords:** crystal structure, amino group, hydrogen bonding, heterocyclic compound

## Abstract

In the title mol­ecule, C_7_H_7_N_3_O, the pyrimidine ring is essentially planar, with the propynyl group rotated out of this plane by 15.31 (4)°. In the crystal, a tri-periodic network is formed by N—H⋯O, N—H⋯N and C—H⋯O hydrogen-bonding and slipped π–π stacking inter­actions, leading to narrow channels extending parallel to the *c* axis. Hirshfeld surface analysis of the crystal structure reveals that the most important contributions for the crystal packing are from H⋯H (36.2%), H⋯C/C⋯H (20.9%), H⋯O/O⋯H (17.8%) and H⋯N/N⋯H (12.2%) inter­actions, showing that hydrogen-bonding and van der Waals inter­actions are the dominant inter­actions in the crystal packing. Evaluation of the electrostatic, dispersion and total energy frameworks indicates that the stabilization is dominated by the electrostatic energy contributions. The mol­ecular structure optimized by density functional theory (DFT) calculations at the B3LYP/6–311 G(d,p) level is compared with the experimentally determined structure in the solid state. The HOMO–LUMO behaviour was also elucidated to determine the energy gap.

## Abstract 2

The mol­ecular structure of the title compound comprises an essentially planar pyrimidine ring from which the propynyl group is rotated by 15.31 (4)°. In the crystal, a tri-periodic network is formed by N—H⋯O, N—H⋯N and C—H⋯O hydrogen-bonding and slipped π–π stacking inter­actions, leading to narrow channels extending parallel to the *c* axis.

## 1. Chemical context

Owing to their importance in the fields of pharmaceuticals, cytosine derivatives and their syntheses have been in the focus of chemists in recent years, in particular during the Covid pandemic period, for example with respect to the synthesis of Molnupiravir as an anti-viral drug (Sahoo & Subba Reddy, 2022 ▸). An alternative product identified as cytarabine, which also has been synthesized from cytosine, is a chemotherapy drug used to treat acute myeloid leukaemia (AML), acute lymphocytic leukaemia (ALL), chronic myeloid leukaemia (CML) and non-Hodgkin’s lymphoma (Lamba, 2009 ▸; Güngör *et al.*, 2022 ▸). 1-(Prop-2-yn­yl)-4-amino-2-oxo­pyrimidine was synthesized as an inter­mediate for the purpose of preparing other products that may have biological activities (Chatzileontiadou *et al.*, 2015 ▸).

In a continuation of our research work devoted to the study of N-alkyl­ation reactions involving cytosine derivatives, we report herein on synthesis, mol­ecular and crystal structures as well as Hirshfeld surface analysis, inter­molecular inter­action energies, energy frameworks and DFT-computational studies of the title compound (I), C_7_H_7_N_3_O. This cytosine derivative was obtained by an alkyl­ation reaction of cytosine using an excess of propargyl bromide as an alkyl­ating reagent under the conditions of phase-transfer catalysis (PTC). 
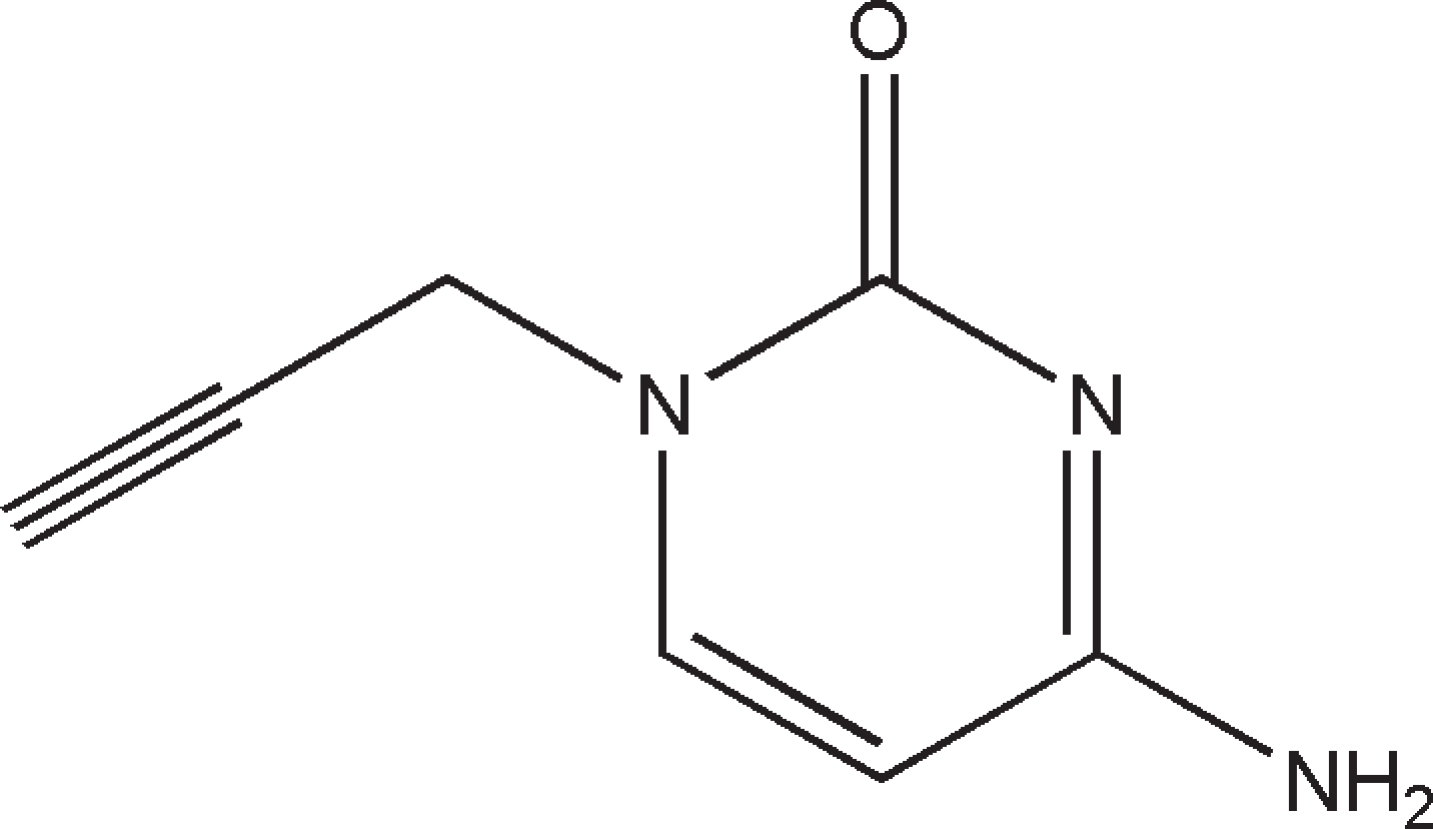


## 2. Structural commentary

**Figure 1:**
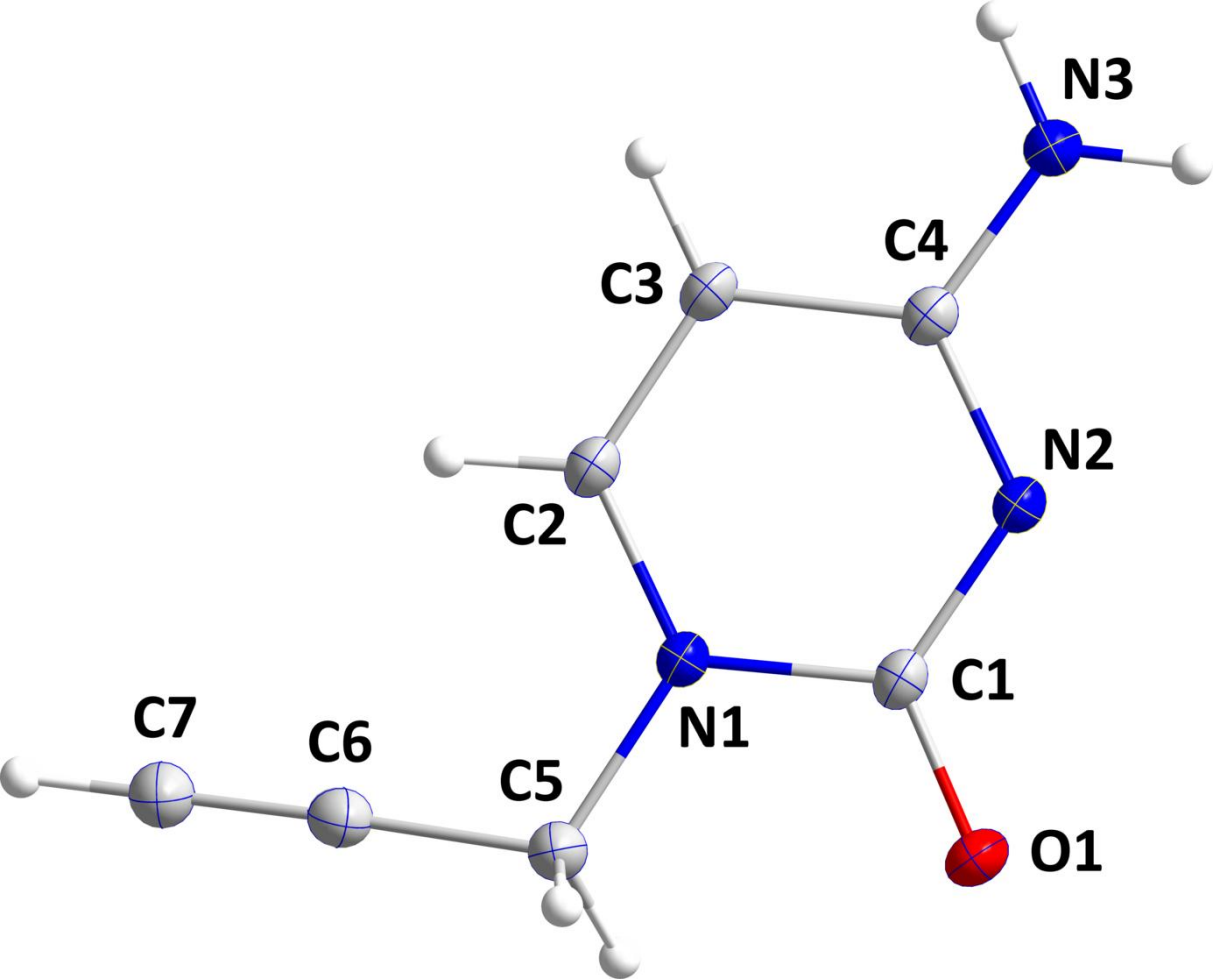
The title mol­ecule with labelling scheme and displacement ellipsoids drawn at the 50% probability level.

The asymmetric unit of (I) comprises one mol­ecule and is shown in Fig. 1 ▸. The pyrimidine ring is essentially planar (r.m.s.d = 0.0055 Å). The plane defined by the propynyl group (N1/C5/C6/C7) is inclined to the pyrimidine plane by 15.31 (4)°.

## 3. Supra­molecular features

**Figure 2:**
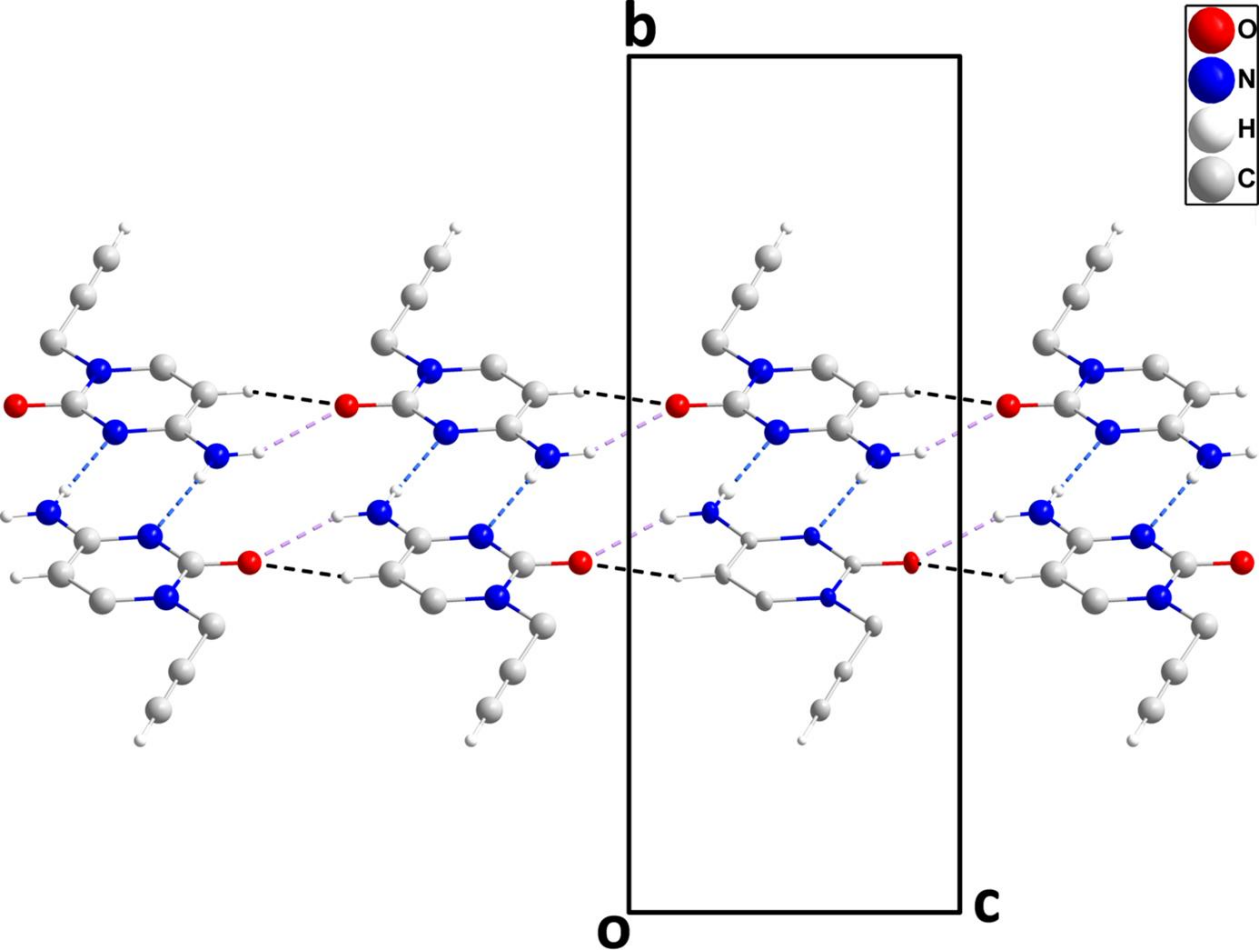
A portion of one ribbon viewed along the *a* axis with N—H⋯N, N—H⋯O and C—H⋯O hydrogen bonds depicted, respectively, by blue, violet and black dashed lines.

**Figure 3:**
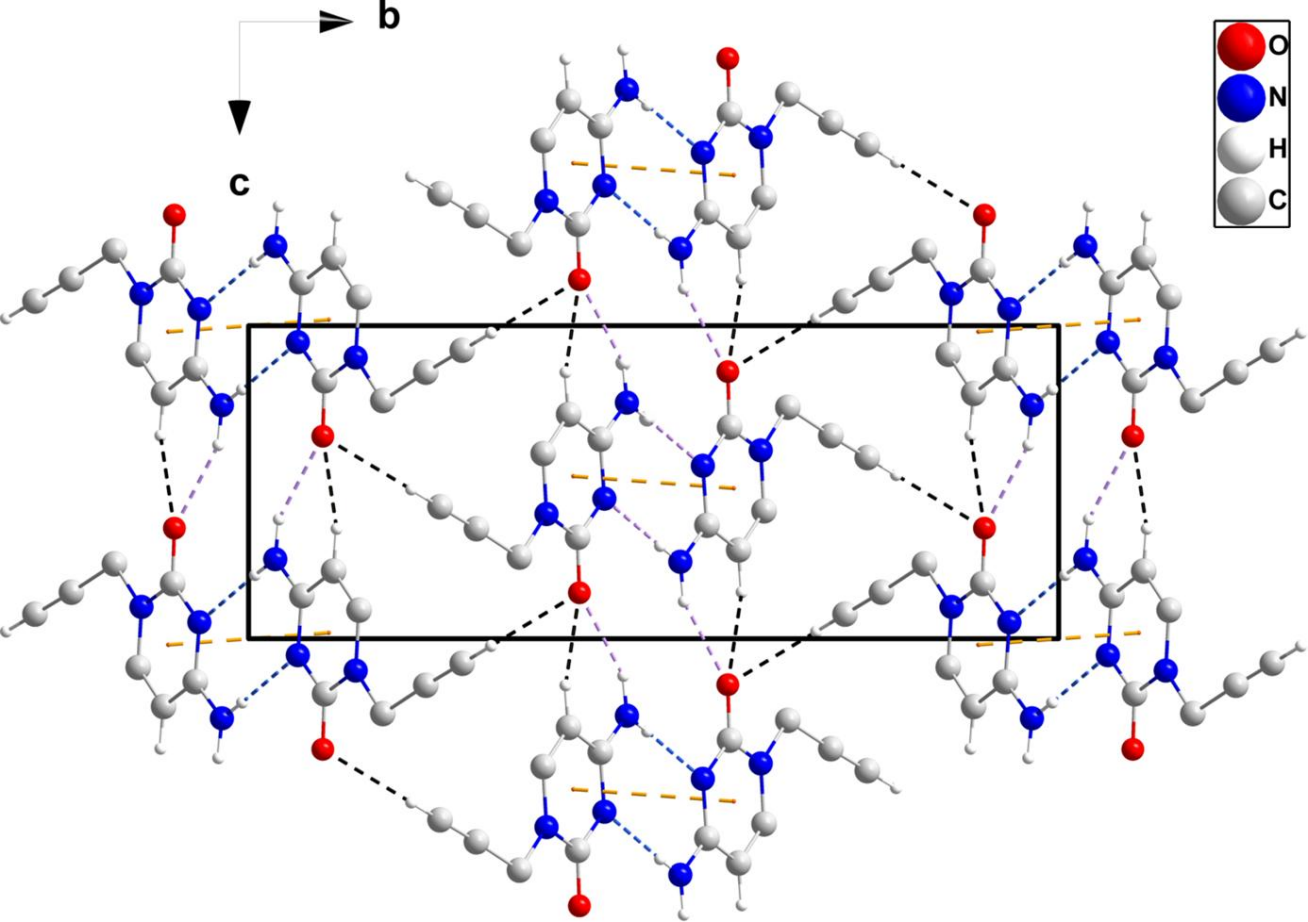
Packing of (I) viewed along the *a* axis with hydrogen bonds depicted as in Fig. 2 ▸. The π–π stacking inter­actions are depicted by orange dashed lines.

**Figure 4:**
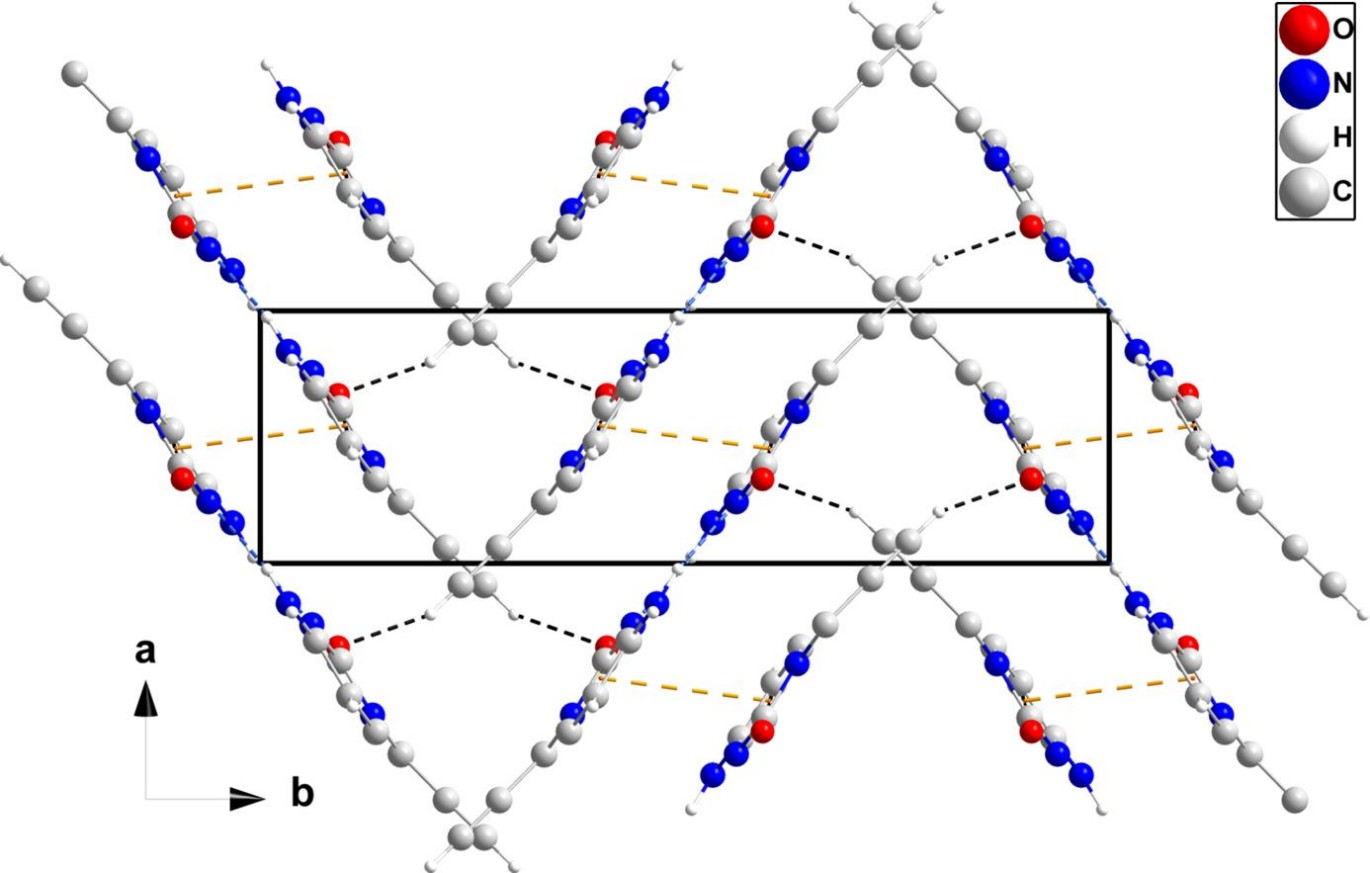
Packing viewed along the *c* axis with hydrogen bonds depicted as in Fig. 2 ▸, and with π–π stacking inter­actions as in Fig. 3 ▸.

In the crystal, N3—H3*A*⋯O1 and C3—H3⋯O1 hydrogen bonds (Table 1 ▸) form chains of mol­ecules extending along the *c*-axis direction. Inversion-related chains are connected by N3—H3*B*⋯N2 hydrogen bonds (Table 1 ▸), forming ribbons whose mean planes are inclined by ±31.4° to (010) (Fig. 2 ▸). The ribbons are linked by C7—H7⋯O1 hydrogen bonds (Table 1 ▸) and slipped π–π stacking inter­actions between pyrimidine rings [centroid-to-centroid distance = 3.6122 (6) Å, slippage = 1.51 Å] into the tri-periodic structure (Fig. 3 ▸), which has narrow channels running parallel to the *c* axis (Fig. 4 ▸).

## 4. Hirshfeld surface analysis

**Figure 5:**
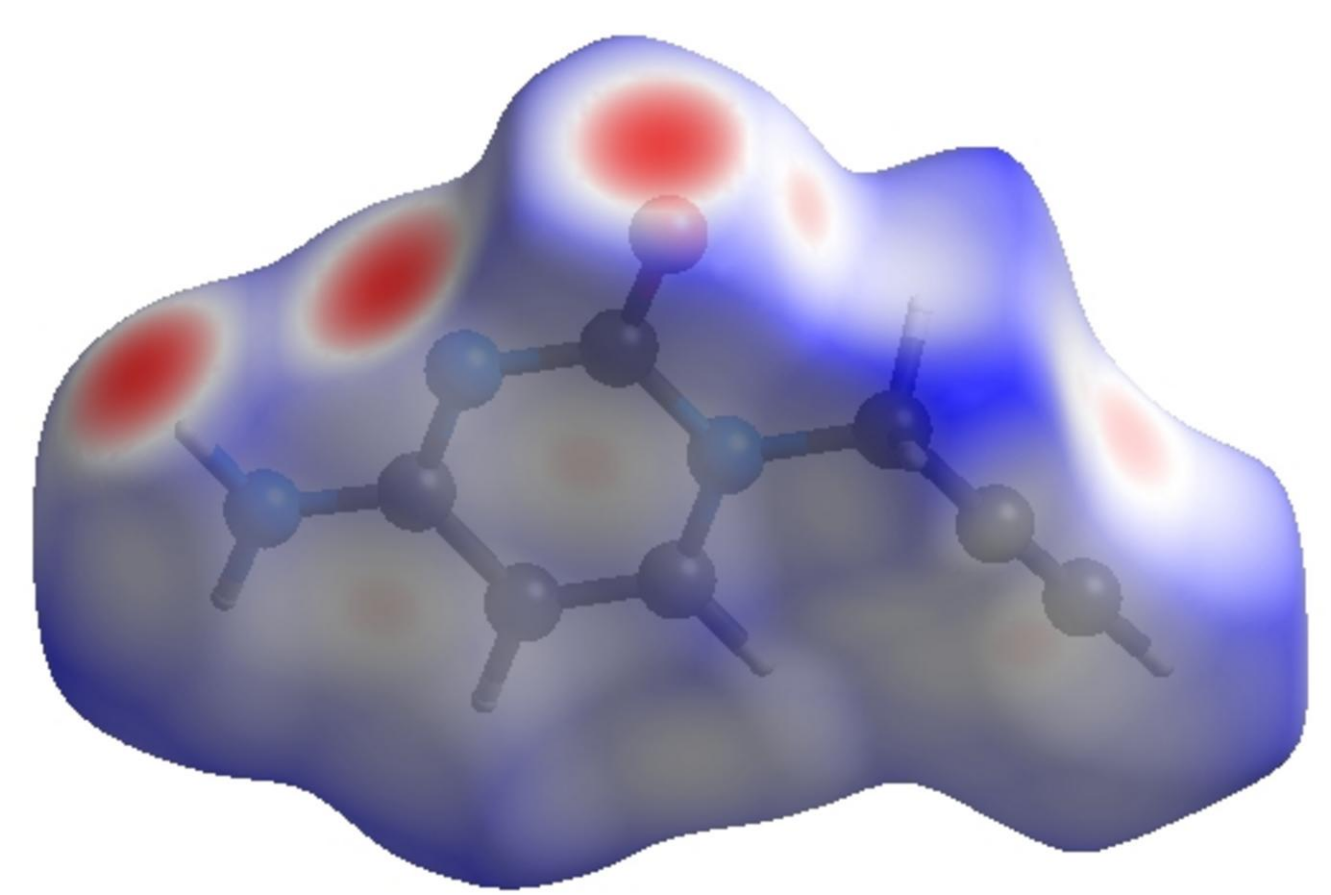
View of the three-dimensional Hirshfeld surface of the title compound plotted over *d*_norm_ in the range of −0.4969 to 1.1244 a.u.

**Figure 6:**
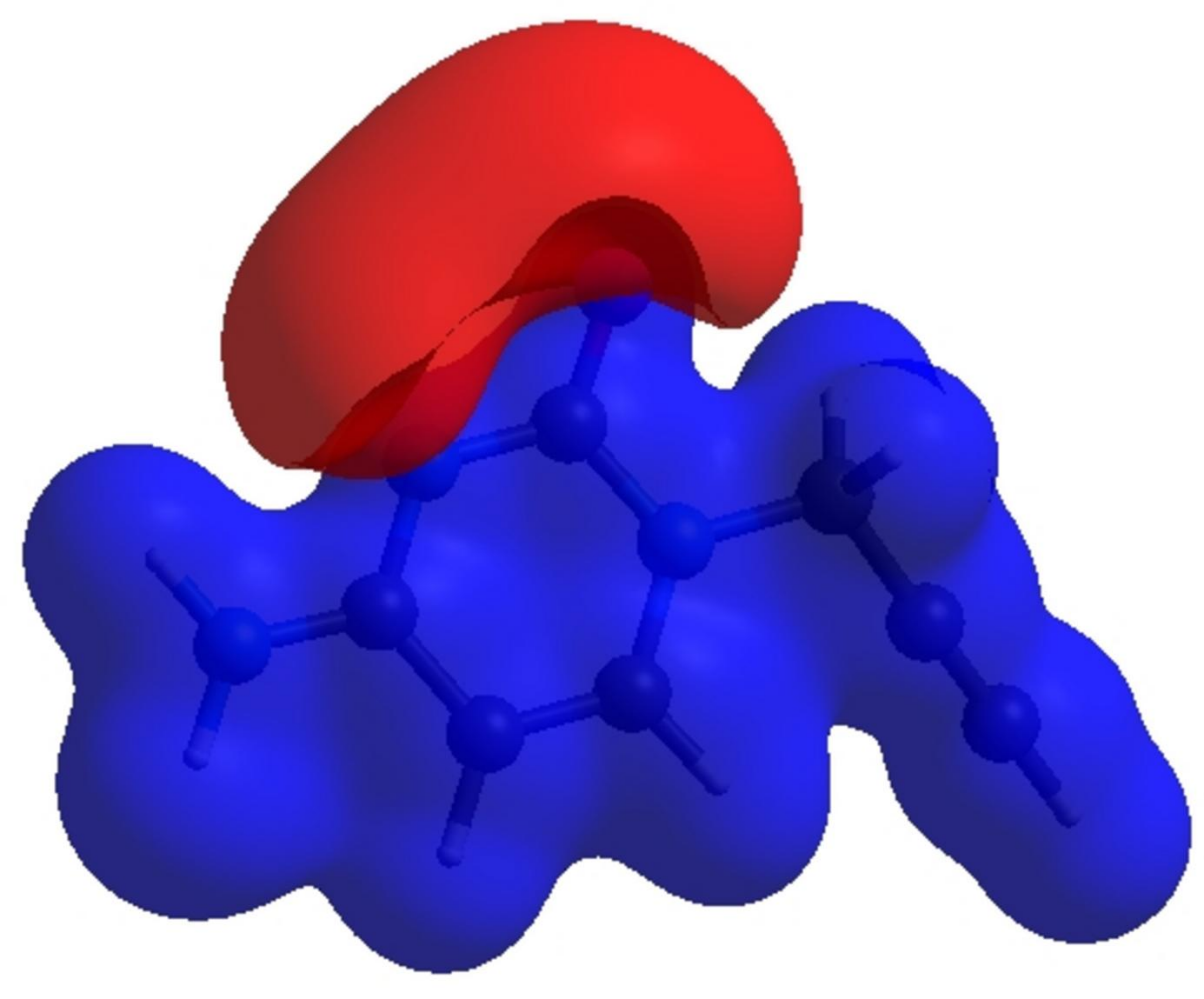
View of the three-dimensional Hirshfeld surface of the title compound plotted over electrostatic potential energy in the range −0.0500 to 0.0500 a.u. using the STO-3 G basis set at the Hartree–Fock level of theory. Hydrogen-bond donors and acceptors are shown as blue and red regions around the atoms, corresponding to positive and negative potentials, respectively.

**Figure 7:**
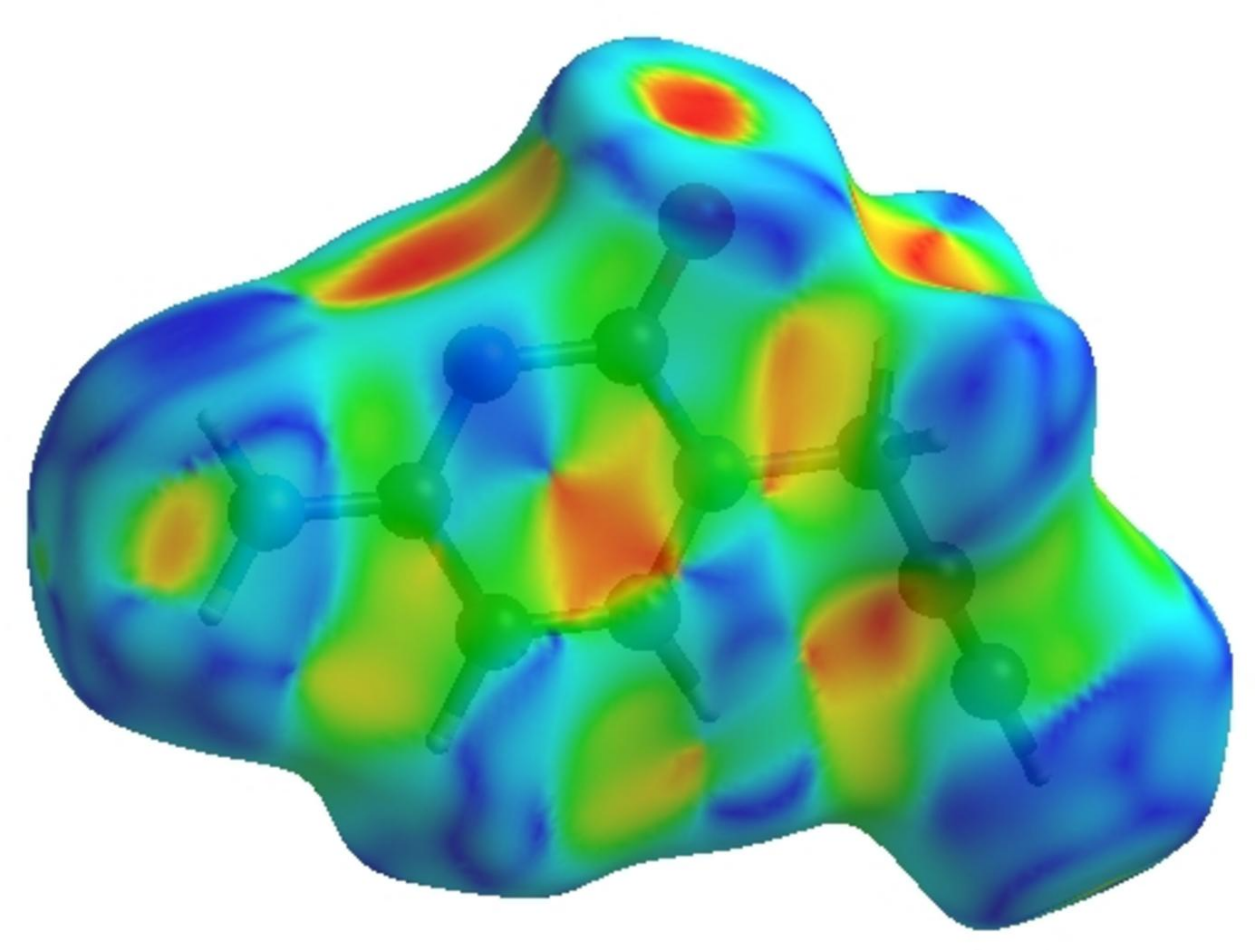
Hirshfeld surface of the title compound plotted over shape-index.

**Figure 8:**
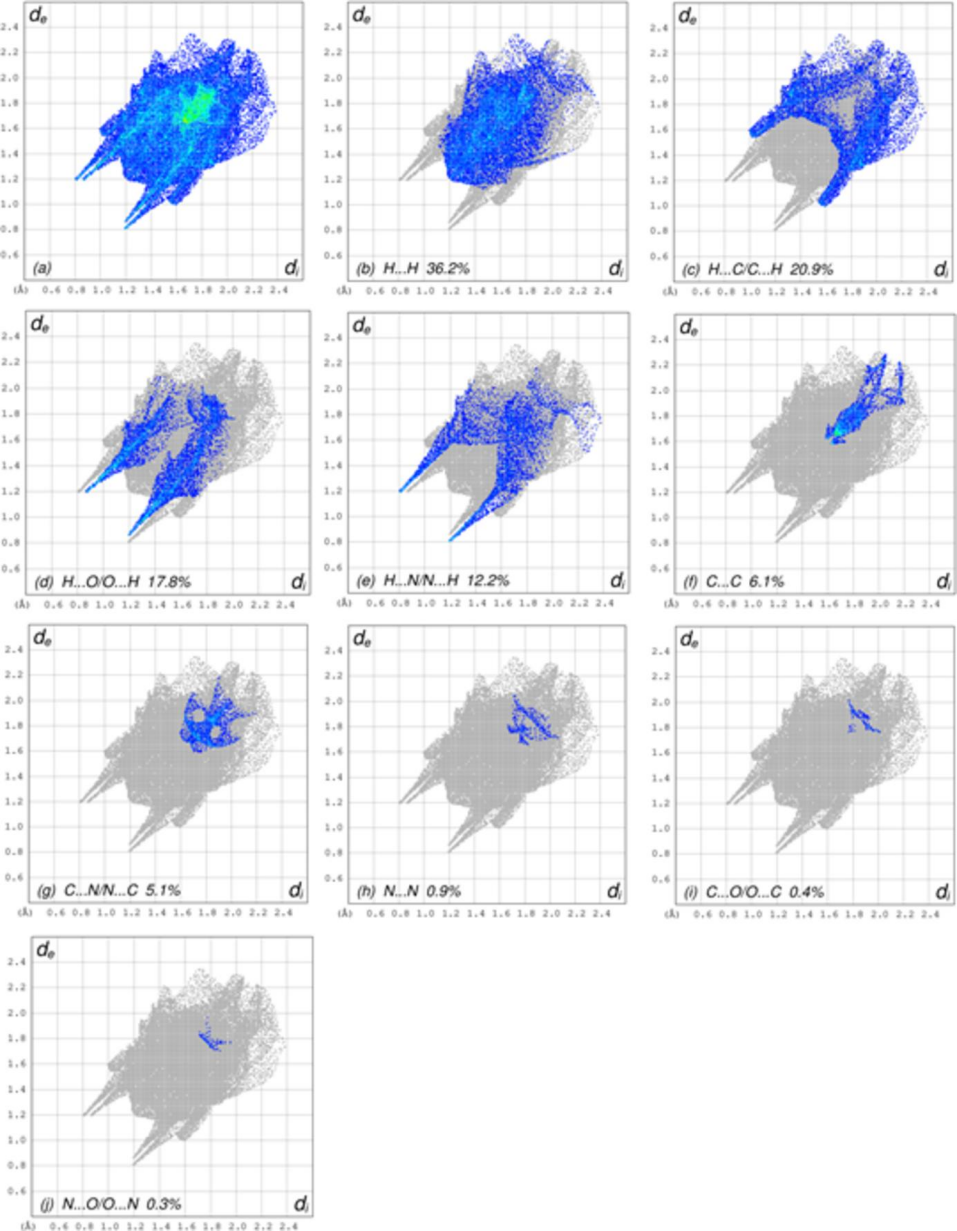
The full two-dimensional fingerprint plots for the title compound, showing (*a*) all inter­actions, and delineated into (*b*) H⋯H, (*c*) H⋯C/C⋯H, (*d*) H⋯O/O⋯H, (*e*) H⋯N/ N⋯H, (*f*) C⋯C, (*g*) C⋯N/N⋯C, (*h*) N⋯N, (i) C⋯O/O⋯C and (*j*) N⋯O/O⋯N inter­actions. The *d*_i_ and *d*_e_ values are the closest inter­nal and external distances (in Å) from given points on the Hirshfeld surface.

**Figure 9:**
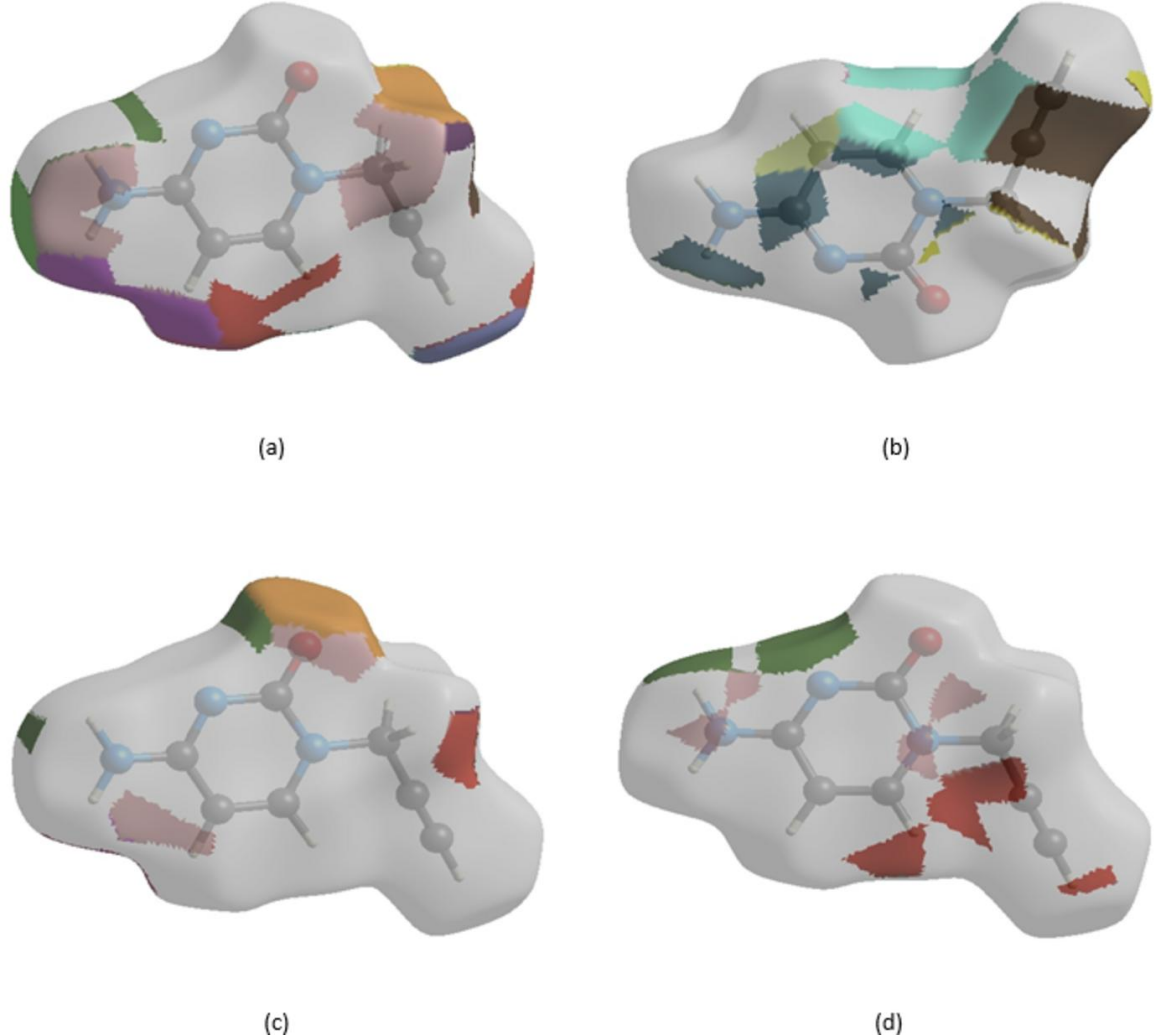
The Hirshfeld surface representations with the function *d*_norm_ plotted onto the surface for (*a*) H⋯H, (*b*) H⋯C/C⋯H, (*c*) H⋯O/O⋯H and (*d*) H⋯N/N⋯H inter­actions.

In order to visualize the inter­molecular inter­actions in the crystal of (I), a Hirshfeld surface (HS) analysis (Hirshfeld, 1977 ▸; Spackman & Jayatilaka, 2009 ▸) was carried out by using *CrystalExplorer* (Spackman *et al.*, 2021 ▸). In the HS plotted over *d*_norm_ (Fig. 5 ▸), the white surface indicates contacts with distances equal to the sum of the van der Waals radii, and the red and blue colours indicate distances shorter (in close contact) or longer (distinct contacts) than the van der Waals radii, respectively (Venkatesan *et al.*, 2016 ▸). The bright-red spots appearing near O1, N2 and hydrogen atom H3*A* indicate their roles as the respective donors and/or acceptors atoms for hydrogen bonding; they also appear as blue and red regions corresponding to positive and negative potentials on the HS mapped over electrostatic potential (Spackman *et al.*, 2008 ▸; Jayatilaka *et al.*, 2005 ▸) shown in Fig. 6 ▸. The blue regions indicate positive electrostatic potential (hydrogen-bond donors), while the red regions indicate negative electrostatic potential (hydrogen-bond acceptors). The shape-index of the HS is a tool to visualize π–π stacking inter­actions by the presence of adjacent red and blue triangles (Fig. 7 ▸). The overall two-dimensional fingerprint plot, Fig. 8 ▸ *a*, and those delineated into H⋯H, H⋯C/C⋯H, H⋯O/O⋯H, H⋯N/ N⋯H, C⋯C, C⋯N/N⋯C, N⋯N, C⋯O/O⋯C and N⋯O/O⋯N (McKinnon *et al.*, 2007 ▸) are illustrated in Fig. 8 ▸ *b*–*j*, together with their relative contributions to the Hirshfeld surface. The most important inter­action originates from H⋯H contacts, contributing 36.2% to the overall crystal packing, which is reflected in Fig. 8 ▸ *b* as widely scattered points of high density due to the large hydrogen content of the mol­ecule with the tip at *d*_e_ = *d*_i_ = 1.20 Å. In the absence of C—H⋯π inter­actions, the H⋯C/C⋯H contacts, contributing 20.9% to the overall crystal packing, are shown in Fig. 8 ▸ *c* with the tips at *d*_e_ + *d*_i_ = 2.57 Å. The pair of characteristic wings in the fingerprint plot delineated into H⋯O/O⋯H contacts (Fig. 8 ▸ *d*) with a 17.8% contribution to the HS is viewed as a pair of spikes with the tips at *d*_e_ + *d*_i_ = 2.05 Å. The pair of characteristic wings in the fingerprint plot delineated into H⋯N/N⋯H contacts (Fig. 8 ▸ *e*, 12.2% contribution to the HS) is viewed as a pair of spikes with the tips at *d*_e_ + *d*_i_ = 2.00 Å. The C⋯C contacts, contributing with 6.1% to the overall crystal packing, have a bullet-shaped distribution of points. They are shown in Fig. 8 ▸ *f* with the tip at *d*_e_ = *d*_i_ = 1.61 Å. The C⋯N/N⋯C contacts,which contribute 5.1% to the overall crystal packing, have a bat-shaped distribution of points (Fig. 8 ▸ *g*) with the tips at *d*_e_ + *d*_i_ = 3.28 Å. Finally, the N⋯N (Fig. 8 ▸ *h*), C⋯O/O⋯C (Fig. 8 ▸ *i*) and N⋯O/O⋯N (Fig. 8 ▸ *j*) contacts contribute 0.9%, 0.4% and 0.3%, respectively, to the HS. The functions *d*_norm_ plotted onto the HS are shown for the H⋯H, H⋯C/C⋯H, H⋯O/O⋯H and H⋯N/N⋯H inter­actions in Fig. 9 ▸ *a*–*d*. The HS analysis confirms the importance of H-atom contacts in establishing the packing and suggest that van der Waals inter­actions and hydrogen-bonding play the major roles in the crystal packing (Hathwar *et al.*, 2015 ▸).

## 5. Inter­action energy calculations and energy frameworks

**Figure 10:**
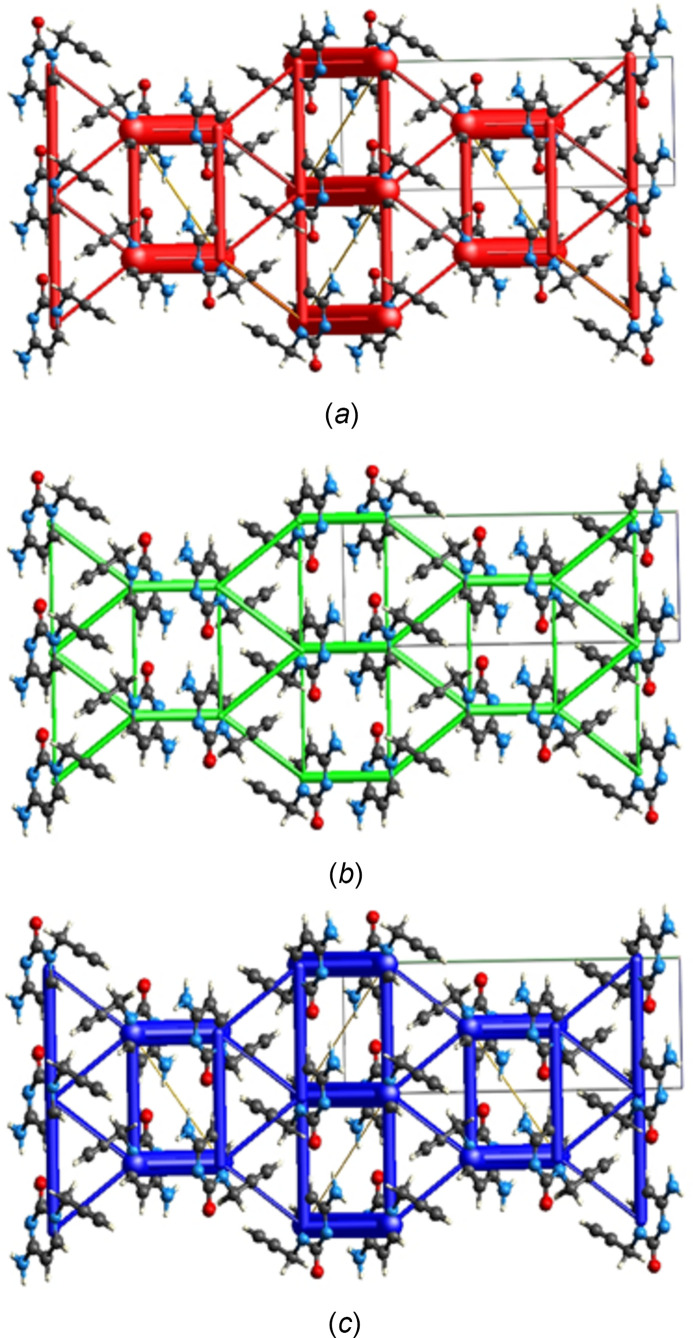
The views of the energy frameworks for a cluster of mol­ecules of the title compound showing (*a*) electrostatic energy, (*b*) dispersion energy and (*c*) total energy diagrams. The cylinder radii are proportional to the relative strength of the corresponding energies, adjusted to the same scale factor of 80 with a cut-off value of 5 kJ mol^−1^ within 2×2×2 unit cells.

The inter­molecular inter­action energies were calculated using the CE–B3LYP/6–31G(d,p) energy model available in *CrystalExplorer* (Spackman *et al.*, 2021 ▸), where a cluster of mol­ecules is generated by applying crystallographic symmetry operations with respect to a selected central mol­ecule within the radius of 3.8 Å by default (Turner *et al.*, 2014 ▸). The total inter­molecular energy (*E*_tot_) is the sum of electrostatic (*E*_ele_), polarization (*E*_pol_), dispersion (*E*_dis_) and exchange-repulsion (*E*_rep_) energies (Turner *et al.*, 2015 ▸) with scale factors of 1.057, 0.740, 0.871 and 0.618, respectively (Mackenzie *et al.*, 2017 ▸). Energy frameworks combine the calculation of inter­molecular inter­action energies with a graphical representation of their magnitude (Turner *et al.*, 2015 ▸). Energies between mol­ecular pairs are represented as cylinders joining the centroids of pairs of mol­ecules with the cylinder radius proportional to the relative strength of the corresponding inter­action energy. Energy frameworks were constructed for *E*_ele_ (red cylinders), *E*_dis_ (green cylinders) and *E*_tot_ (blue cylinders) and are shown in Fig. 10 ▸ *a–c*. The evaluation of the electrostatic, dispersion and total energy frameworks reveals that the stabilization is dominated by the electrostatic energy contribution in the crystal structure of (I).

## 6. DFT calculations

**Figure 11:**
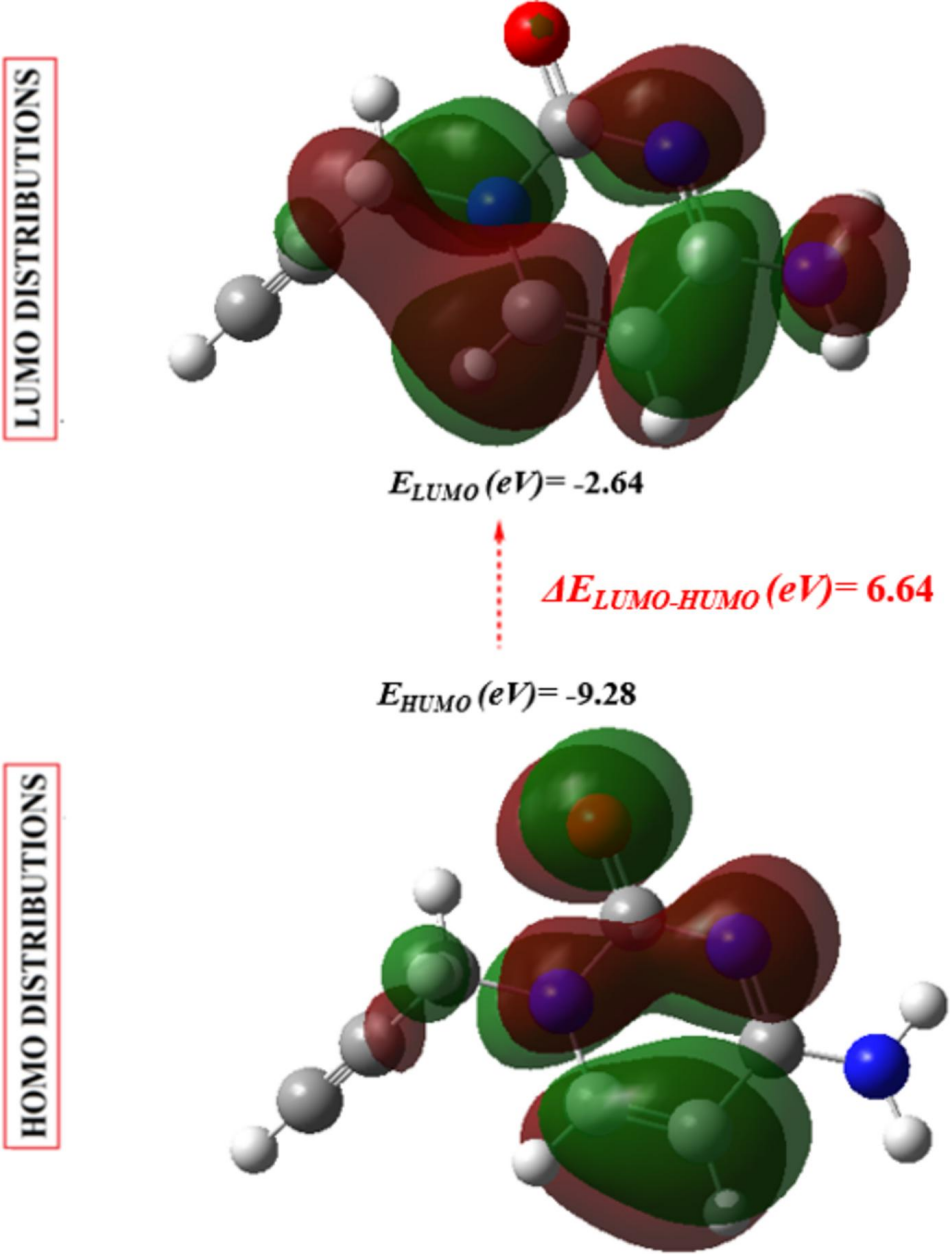
The energy band gap of (I).

Bond lengths and angles as well as energies of (I) in the gas phase were computed on basis of density functional theory (DFT) using the standard B3LYP functional and the 6–311G(d,p) basis-set (Becke, 1993 ▸) as implemented in *GAUSSIAN 09* (Frisch *et al.*, 2009 ▸). Table 2 ▸ reveals that the calculated bond lengths and angles are in good agreement with the experimentally determined values. The HOMO-LUMO energy gap of the mol­ecule was also calculated by the DFT/B3LYP/6-311G(d,p) method and is shown in Fig. 11 ▸. Furthermore, quantum chemistry descriptors (chemical hardness *η*, softness *S*, electronegativity *χ* and electrophilicity *w*) derived from the conceptual DFT calculations of (I) are given in Table 3 ▸. The HOMO and LUMO are localized in the plane extending from the whole 4-amino-1-(prop-2-yn-1-yl)pyrimidin-2(1*H*)-one ring. The energy band gap [Δ*E* = *E*_LUMO_ − *E*_HOMO_] of the mol­ecule is 6.64 eV, and the frontier mol­ecular orbital energies, *E*_HOMO_ and *E*_LUMO_ are −9.28 eV and −2.64 eV, respectively.

## 7. Database survey

**Figure 12:**
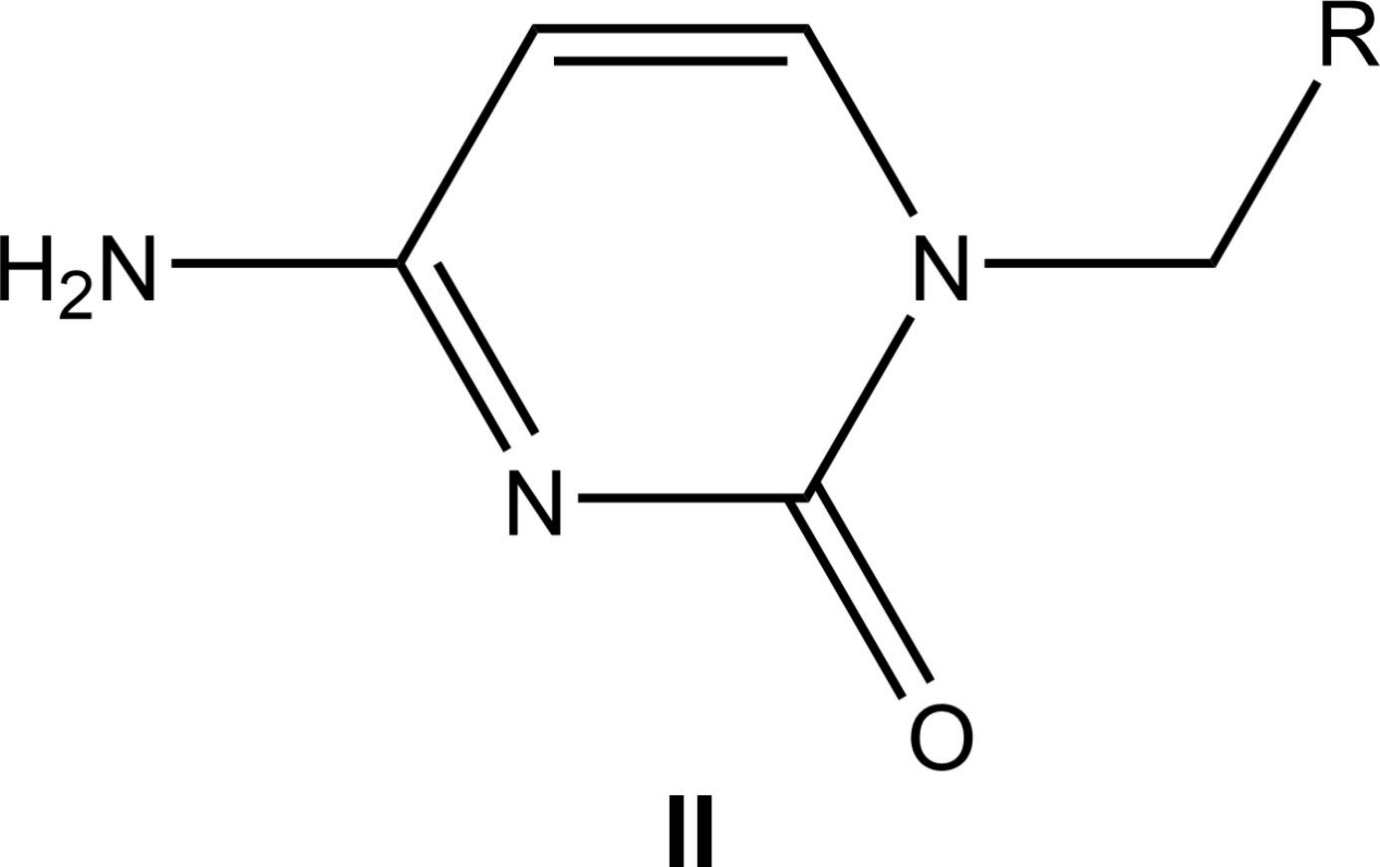
The mol­ecular fragment **II** used for the database search.

A search of the Cambridge Structural Database (CSD, version 5.42, current as of October 2023; Groom *et al.*, 2016 ▸) with the search fragment **II** (Fig. 12 ▸) generated 37 hits also including co-crystals, metal complexes and ions protonated on the doubly bonded nitro­gen atom. The most comparable structures to (I) include those with *R* = CH_2_CO_2_Bu^i^ (COFJIS; Geng *et al.*, 2013 ▸), 2-oxido-2-phen­oxy-1,4,2-dioxaphosphinan-5-yl (DATZIJ; Krylov *et al.*, 2012 ▸), CH_2_C(=O)NHC(COOH)CH_2_(4-OHC_6_H_4_) (COQNAX; Doi *et al.*, 1999 ▸), (CH_2_)_3_SiMe_2_Ph (HIXKOQ; Kociok-Köhn *et al.*, 2014 ▸), CH=CHCH_2_NHC(=O)(4-FC_6_H_4_) (PEYHOS; Cetina *et al.*, 2012 ▸), CH_2_OH (DECYUF; Shibata *et al.*, 1985 ▸), CH_2_C(=O)NH_2_ (CIMJEN; Fujita *et al.*, 1984 ▸), CH_2_C(=O)NHC(COO^−^)(CH_2_)_4_NH_3_^+^ (LAVZEO; Doi *et al.*, 2005 ▸), 2′-de­oxy-β-d-ribo-pento­furanosyl (NAGLIQ; Hossain *et al.*, 1996 ▸), 4-pyridyl (KUDPEH; Tufenkjian *et al.*, 2020 ▸), CH=CHCH_2_NHC(O)Ph (PEHYEI; Cetina *et al.*, 2012 ▸) and *n*-pentyl (YINGAF; Barceló-Oliver *et al.*, 2013 ▸). In all of these structures, the first two atoms of the substituent are rotated by nearly 90° from being coplanar with the pyrimidine ring, in contrast to what is observed for (I). In all cases this is likely due to steric hindrance between hydrogen atoms on the substituent and the adjacent ring hydrogen and the carbonyl oxygen. However, in COQNAX there is a possible, weak π-stacking inter­action that could also direct the conformation. In NAGLIQ, there is a weak C—H⋯π(ring) inter­action that could act similarly.

## 8. Synthesis and crystallization

A mixture of cytosine (1.5 mmol) and potassium carbonate (K_2_CO_3_) (3 mmol) was dissolved in 25 ml of di­methyl­formamide (DMF). The solution was stirred magnetically for 10 min., followed by addition of 0.01 equivalents of tetra-*n*-butyl­ammonium bromide (TBAB) and 3 mmol of propargyl bromide. The mixture was stirred magnetically for 24 h. After filtration of the formed salts, the DMF was evaporated under reduced pressure. The residue obtained was purified by chromatography on a silica gel column. Single crystals of (I) suitable for X-ray diffraction were obtained by slow evaporation of an ethanol solution. ^1^H NMR (300 MHz, DMSO-*d*_6_): 3.306–3.322 (*t*, 1H, CH≡C, *J* = 2.4, 4.8); 4.482–4.49 (*d*, 2H, CH_2_, *J* = 2.4); 5.806–5.83 (*d*, 1H, CH, *J* = 7.2); 7.109 (*s*, 1H, NH); 7.37 (*s*, 1H, NH); 7.669–7.693 (*d*, 1H, CH—N, *J* = 7.2). ^13^C NMR (75 MHz, DMSO): 37.95 (CH_2_); 75.78 (C≡CH); 94.93 (CH≡C); 141.48 (CH—N); 143.24 (CH—C); 156.05 (C=O); 166.42 (C=N).

## 9. Refinement

Crystal data, data collection and structure refinement details are summarized in Table 4 ▸. H atoms attached to carbon atoms were placed in idealized positions and were included as riding contributions with isotropic displacement parameters 1.2–1.5 times those of the parent atoms. Those attached to nitro­gen were placed in locations derived from a difference-Fourier map and refined with a distance of 0.90 (1) Å. Reflection 020 was affected by the beamstop and was omitted from the final refinement.

## Supplementary Material

Crystal structure: contains datablock(s) global, I. DOI: 10.1107/S2056989023009933/wm5702sup1.cif

Structure factors: contains datablock(s) I. DOI: 10.1107/S2056989023009933/wm5702Isup2.hkl

Supporting information file. DOI: 10.1107/S2056989023009933/wm5702Isup3.cdx

Supporting information file. DOI: 10.1107/S2056989023009933/wm5702Isup4.cml

CCDC reference: 2308262

Additional supporting information:  crystallographic information; 3D view; checkCIF report

## Figures and Tables

**Table 1:** Hydrogen-bond geometry (Å, °)

*D*—H⋯*A*	*D*—H	H⋯*A*	*D*⋯*A*	*D*—H⋯*A*
N3—H3*A*⋯O1^i^	0.89 (1)	2.16 (1)	3.0002 (9)	156 (1)
N3—H3*B*⋯N2^ii^	0.90 (1)	2.10 (1)	2.9854 (10)	169 (1)
C3—H3⋯O1^i^	0.95	2.56	3.3036 (10)	135
C7—H7⋯O1^iii^	0.95	2.37	3.2559 (11)	156

Symmetry codes: (i) 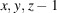 ; (ii) 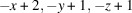 ; (iii) 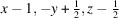 .

**Table 2:** Comparison of selected X-ray and DFT bond lengths and angles (Å, °)

Bonds/angles	X-ray	B3LYP/6–311G(d,p)
O1—C1	1.2444 (9)	1.2457
N1—C2	1.3632 (10)	1.3645
N1—C5	1.4716 (10)	1.4782
N2—C4	1.3415 (10)	1.3423
N2—C1	1.3570 (10)	1.3542
N3—C4	1.3353 (10)	1.3392
C2—N1—C1	120.71 (6)	120.82
O1—C1—N2	122.47 (7)	122.17
O1—C1—N1	118.64 (7)	118.54
N2—C1—N1	118.88 (6)	118.53

**Table 3:** Calculated energies and quantum-chemical parameters of (I)

Total Energy, *TE* (eV)	−13800.94
*E*_HOMO_ (eV)	−9.28
*E*_LUMO_ (eV)	−2.64
Gap, *ΔE* (eV)	6.64
Dipole moment, *μ* (Debye)	7.47
Ionization potential, *I* (eV)	9.28
Electron affinity, *A*	2.64
Electronegativity, *χ*	3.20
Hardness, *η*	5.96
Softness, *σ*	0.15
Electrophilicity index, *ω*	5.35

**Table 4:** Experimental details

Crystal data	
Chemical formula	C_7_H_7_N_3_O
*M*_r_	149.16
Crystal system, space group	Monoclinic, *P*2_1_/*c*
Temperature (K)	150
*a*, *b*, *c* (Å)	5.3864 (6), 18.013 (2), 7.0112 (8)
β (°)	96.288 (4)
*V* (Å^3^)	676.18 (13)
*Z*	4
Radiation type	Mo *K*α
μ (mm^−1^)	0.10
Crystal size (mm)	0.34 × 0.32 × 0.07

Data collection	
Diffractometer	Bruker D8 QUEST PHOTON 3 diffractometer
Absorption correction	Numerical (*SADABS*; Krause *et al.*, 2015 ▸)
*T*_min_, *T*_max_	0.96, 0.99
No. of measured, independent and observed [*I* > 2σ(*I*)] reflections	28024, 2554, 2251
*R*_int_	0.035
(sin θ/λ)_max_ (Å^−1^)	0.770

Refinement	
*R*[*F* ^2^ > 2σ(*F* ^2^)], *wR*(*F* ^2^), *S*	0.040, 0.113, 1.05
No. of reflections	2554
No. of parameters	108
No. of restraints	2
H-atom treatment	H atoms treated by a mixture of independent and constrained refinement
Δρ_max_, Δρ_min_ (e Å^−3^)	0.47, −0.20

Computer programs: *APEX3* and *SAINT* (Bruker, 2020 ▸), *SHELXT* (Sheldrick, 2015*a* ▸), *SHELXL* (Sheldrick, 2015*b* ▸), *DIAMOND* (Brandenburg & Putz, 2012 ▸) and *publCIF* (Westrip, 2010 ▸).

## supplementary crystallographic information

### Crystal data

**Table:**

C_7_H_7_N_3_O	*F*(000) = 312
*M**_r_* = 149.16	*D*_x_ = 1.465 Mg m^−^^3^
Monoclinic, *P*2_1_/*c*	Mo *K*α radiation, λ = 0.71073 Å
*a* = 5.3864 (6) Å	Cell parameters from 9914 reflections
*b* = 18.013 (2) Å	θ = 4.0–33.2°
*c* = 7.0112 (8) Å	µ = 0.10 mm^−^^1^
β = 96.288 (4)°	*T* = 150 K
*V* = 676.18 (13) Å^3^	Plate, colourless
*Z* = 4	0.34 × 0.32 × 0.07 mm

### Data collection

**Table:**

Bruker D8 QUEST PHOTON 3 diffractometer	2554 independent reflections
Radiation source: fine-focus sealed tube	2251 reflections with *I* > 2σ(*I*)
Graphite monochromator	*R*_int_ = 0.035
Detector resolution: 7.3910 pixels mm^-1^	θ_max_ = 33.2°, θ_min_ = 4.0°
ω scans	*h* = −8→8
Absorption correction: numerical (*SADABS*; Krause *et al.*, 2015)	*k* = −27→27
*T*_min_ = 0.96, *T*_max_ = 0.99	*l* = −10→10
28024 measured reflections	

### Refinement

**Table:**

Refinement on *F*^2^	Primary atom site location: dual
Least-squares matrix: full	Secondary atom site location: difference Fourier map
*R*[*F*^2^ > 2σ(*F*^2^)] = 0.040	Hydrogen site location: mixed
*wR*(*F*^2^) = 0.113	H atoms treated by a mixture of independent and constrained refinement
*S* = 1.05	*w* = 1/[σ^2^(*F*_o_^2^) + (0.0627*P*)^2^ + 0.1628*P*] where *P* = (*F*_o_^2^ + 2*F*_c_^2^)/3
2554 reflections	(Δ/σ)_max_ < 0.001
108 parameters	Δρ_max_ = 0.47 e Å^−^^3^
2 restraints	Δρ_min_ = −0.20 e Å^−^^3^

### Special details

**Table:**

Experimental. The diffraction data were obtained from 8 sets of frames, each of width 0.5° in ω, collected with scan parameters determined by the "strategy" routine in *APEX3*. The scan time was 5 sec/frame.
Geometry. All esds (except the esd in the dihedral angle between two l.s. planes) are estimated using the full covariance matrix. The cell esds are taken into account individually in the estimation of esds in distances, angles and torsion angles; correlations between esds in cell parameters are only used when they are defined by crystal symmetry. An approximate (isotropic) treatment of cell esds is used for estimating esds involving l.s. planes.
Refinement. Refinement of F^2^ against ALL reflections. The weighted R-factor wR and goodness of fit S are based on F^2^, conventional R-factors R are based on F, with F set to zero for negative F^2^. The threshold expression of F^2^ > 2sigma(F^2^) is used only for calculating R-factors(gt) etc. and is not relevant to the choice of reflections for refinement. R-factors based on F^2^ are statistically about twice as large as those based on F, and R- factors based on ALL data will be even larger. H-atoms attached to carbon were placed in calculated positions (C—H = 0.95 - 1.00 Å) and were included as riding contributions with isotropic displacement parameters 1.2 - 1.5 times those of the attached atoms. Those attached to nitrogen were placed in locations derived from a difference map and refined with a DFIX 0.91 0.01 instruction. One reflection affected by the beamstop was omitted from the final refinement.

### Fractional atomic coordinates and isotropic or equivalent isotropic displacement parameters (Å^2^)

**Table:**

	*x*	*y*	*z*	*U*_iso_*/*U*_eq_	
O1	0.66696 (11)	0.40841 (3)	0.85317 (8)	0.01939 (14)	
N1	0.39821 (12)	0.36777 (4)	0.60167 (9)	0.01532 (14)	
N2	0.75623 (12)	0.43923 (3)	0.55258 (9)	0.01525 (14)	
N3	0.83960 (13)	0.46704 (4)	0.24590 (10)	0.01878 (15)	
H3A	0.805 (3)	0.4626 (8)	0.1187 (12)	0.031 (3)*	
H3B	0.976 (2)	0.4921 (7)	0.296 (2)	0.033 (3)*	
C1	0.61327 (14)	0.40625 (4)	0.67606 (11)	0.01469 (14)	
C2	0.33252 (14)	0.36368 (4)	0.40859 (11)	0.01677 (15)	
H2	0.183831	0.338315	0.361154	0.020*	
C3	0.47496 (14)	0.39504 (4)	0.28349 (11)	0.01730 (15)	
H3	0.431950	0.391252	0.148775	0.021*	
C4	0.69319 (14)	0.43416 (4)	0.36256 (11)	0.01446 (14)	
C5	0.24325 (15)	0.33434 (4)	0.73922 (11)	0.01883 (16)	
H5A	0.352977	0.308719	0.841101	0.023*	
H5B	0.153456	0.374207	0.800556	0.023*	
C6	0.06137 (15)	0.28125 (4)	0.64860 (11)	0.01895 (16)	
C7	−0.08594 (16)	0.23629 (5)	0.57853 (12)	0.02220 (17)	
H7	−0.202500	0.200721	0.523077	0.027*	

### Atomic displacement parameters (Å^2^)

**Table:**

	*U*^11^	*U*^22^	*U*^33^	*U*^12^	*U*^13^	*U*^23^
O1	0.0215 (3)	0.0237 (3)	0.0123 (3)	−0.0033 (2)	−0.0012 (2)	−0.00096 (19)
N1	0.0150 (3)	0.0178 (3)	0.0128 (3)	−0.0033 (2)	0.0001 (2)	−0.0006 (2)
N2	0.0154 (3)	0.0169 (3)	0.0130 (3)	−0.0025 (2)	−0.0001 (2)	−0.0009 (2)
N3	0.0190 (3)	0.0232 (3)	0.0140 (3)	−0.0051 (2)	0.0011 (2)	0.0000 (2)
C1	0.0142 (3)	0.0153 (3)	0.0140 (3)	−0.0008 (2)	−0.0008 (2)	−0.0015 (2)
C2	0.0161 (3)	0.0194 (3)	0.0142 (3)	−0.0028 (2)	−0.0009 (2)	−0.0017 (2)
C3	0.0177 (3)	0.0207 (3)	0.0130 (3)	−0.0034 (2)	−0.0008 (2)	−0.0023 (2)
C4	0.0145 (3)	0.0148 (3)	0.0138 (3)	0.0002 (2)	0.0003 (2)	−0.0010 (2)
C5	0.0195 (3)	0.0218 (3)	0.0151 (3)	−0.0051 (3)	0.0014 (3)	0.0004 (2)
C6	0.0192 (3)	0.0206 (3)	0.0170 (3)	−0.0014 (3)	0.0019 (3)	0.0025 (3)
C7	0.0227 (4)	0.0240 (4)	0.0195 (4)	−0.0044 (3)	0.0007 (3)	0.0015 (3)

### Geometric parameters (Å, º)

**Table:**

O1—C1	1.2444 (9)	C2—C3	1.3507 (11)
N1—C2	1.3632 (10)	C2—H2	0.9500
N1—C1	1.4007 (10)	C3—C4	1.4294 (10)
N1—C5	1.4716 (10)	C3—H3	0.9500
N2—C4	1.3415 (10)	C5—C6	1.4635 (11)
N2—C1	1.3570 (10)	C5—H5A	0.9900
N3—C4	1.3353 (10)	C5—H5B	0.9900
N3—H3A	0.894 (8)	C6—C7	1.2005 (11)
N3—H3B	0.899 (9)	C7—H7	0.9500
			
C2—N1—C1	120.71 (6)	C2—C3—H3	121.4
C2—N1—C5	121.63 (6)	C4—C3—H3	121.4
C1—N1—C5	117.63 (6)	N3—C4—N2	118.42 (7)
C4—N2—C1	120.28 (6)	N3—C4—C3	119.79 (7)
C4—N3—H3A	120.0 (9)	N2—C4—C3	121.78 (7)
C4—N3—H3B	119.5 (10)	C6—C5—N1	112.56 (6)
H3A—N3—H3B	120.4 (13)	C6—C5—H5A	109.1
O1—C1—N2	122.47 (7)	N1—C5—H5A	109.1
O1—C1—N1	118.64 (7)	C6—C5—H5B	109.1
N2—C1—N1	118.88 (6)	N1—C5—H5B	109.1
C3—C2—N1	121.21 (7)	H5A—C5—H5B	107.8
C3—C2—H2	119.4	C7—C6—C5	178.11 (9)
N1—C2—H2	119.4	C6—C7—H7	180.0
C2—C3—C4	117.12 (7)		
			
C4—N2—C1—O1	178.71 (7)	N1—C2—C3—C4	−1.57 (11)
C4—N2—C1—N1	−0.74 (10)	C1—N2—C4—N3	−179.62 (7)
C2—N1—C1—O1	−179.75 (7)	C1—N2—C4—C3	0.61 (11)
C5—N1—C1—O1	2.17 (10)	C2—C3—C4—N3	−179.22 (7)
C2—N1—C1—N2	−0.27 (11)	C2—C3—C4—N2	0.55 (11)
C5—N1—C1—N2	−178.35 (6)	C2—N1—C5—C6	15.87 (11)
C1—N1—C2—C3	1.48 (12)	C1—N1—C5—C6	−166.07 (7)
C5—N1—C2—C3	179.48 (7)		

### Hydrogen-bond geometry (Å, º)

**Table:**

*D*—H···*A*	*D*—H	H···*A*	*D*···*A*	*D*—H···*A*
N3—H3*A*···O1^i^	0.89 (1)	2.16 (1)	3.0002 (9)	156 (1)
N3—H3*B*···N2^ii^	0.90 (1)	2.10 (1)	2.9854 (10)	169 (1)
C3—H3···O1^i^	0.95	2.56	3.3036 (10)	135
C7—H7···O1^iii^	0.95	2.37	3.2559 (11)	156

Symmetry codes: (i) *x*, *y*, *z*−1; (ii) −*x*+2, −*y*+1, −*z*+1; (iii) *x*−1, −*y*+1/2, *z*−1/2.
